# Influential Factors of Insufficient Physical Activity among Adolescents with Asthma in Taiwan

**DOI:** 10.1371/journal.pone.0116417

**Published:** 2014-12-31

**Authors:** Yu-Kuei Teng, Jing-Long Huang, Kuo-Wei Yeh, Lin-Shien Fu, Chia-Huei Lin, Wei-Fen Ma, Shin-Da Lee, Li-Chi Chiang

**Affiliations:** 1 Graduate Institute of Clinical Medical Science, China Medical University, Taichung, Taiwan; 2 School of Nursing, China Medical University, Taichung, Taiwan; 3 Division of Allergy Asthma and Rheumatology, Department of Pediatrics, Chang Gung Memorial Hospital, and Chang Gung University, Taoyuan, Taiwan; 4 Division of Pediatric Immunology and Nephrology, Department of Pediatrics, Taichung Veterans General Hospital, Taichung, Taiwan; 5 Graduate Institute of Medical Science, Taipei, Taiwan; 6 School of Nursing, National Defense Medical Center, Taipei, Taiwan; 7 Department of Nursing, Tri-Service General Hospital, Taipei, Taiwan; 8 Department of Physical Therapy, Graduate Institute of Rehabilitation Science, China Medical University, Taichung, Taiwan; 9 Department of Healthcare Administration, Asia University, Taichung, Taiwan; West Virginia University School of Medicine, United States of America

## Abstract

**Purpose:**

Little research has been reported concerning insufficient physical activity in Taiwanese adolescents with asthma. The aims of this paper are to compare the amount of physical activity between asthmatic and non-asthmatic adolescents in Taiwan, as well as to investigate the influential factors associated with insufficient physical activity in asthmatic adolescents.

**Methods:**

Self-reporting structured questionnaires (socio-economic status, scale of family support for physical activity, amount of physical activity) and peak expiratory flow were assessed from 286 adolescents with asthma and 588 non-asthmatic adolescents in a cross-sectional design. Insufficient amount of physical activity was based on less than 300 minutes per week of moderate and vigorous physical activity.

**Results:**

Adolescents with asthma have a greater amount of physical activity and a higher level of family support than those who are non-asthmatic. In Taiwan, adolescents with asthma, girls relative to boys, obesity relative to average weight, and low family support relative to high family support were found to be associated with insufficient physical activity.

**Conclusion:**

Physical activity in adolescents with asthma is insufficient especially in girls, in asthmatics with obesity, and in those with low family support. We suggest that physical activity programs should be applied to Taiwan adolescents with asthma in order to match the criteria of 300 minutes per week of moderate and vigorous physical activity, especially for girls, the obese and those with a low level of family support.

## Introduction

Physical inactivity is known to be fourth leading risk factor for global mortality and has a major influence on the prevalence of non-communicable diseases and the general health of the population worldwide [1]. In a systematic review, the prevalence of insufficient physical activity varied from 18.7 to 90.6% of adolescents by the definition of insufficient physical activity as being less than 300 minutes per week of moderate and vigorous physical activity (based on a cutoff of less than 60 minutes per day and 5 days per week as an insufficient level of physical activity) [2]. It has been reported in the literature that decreased physical activity is a contributor to the increase in asthma prevalence and severity [3], [4]. However, little has been reported concerning insufficient physical activity based on less than 300 minutes per week of moderate and vigorous physical activity in Taiwanese adolescents with asthma [5].

Children with asthma were found to have lower average daily physical activity (asthma 116 minutes vs. non-asthma 146 minutes) and their daily physical activity tended to be less than 30 minutes per day (asthma 21% vs. non-asthma 9%) compared to children with non-asthma [6]. On the other hand, children with asthma were not significantly different in moderate to vigorous physical activity compared to children without asthma [7] and a study has reported that they had less sedentary time but still had asthma [4]. Rasmussen et al. found that a decrease in the levels of physical activity of children was correlated with the increased risk of adolescent asthma [3]. A significant improvement in peak expiratory flow was found when children with asthma increased their physical activities [8]. Well-controlled asthma was associated with relevant increased daily amount of physical activity and cardiovascular fitness [9]. As a summary result, physical activity habits are important for living with asthma. It is still unknown how far from 300 minutes physical activity per week are Taiwan adolescents with asthma.

Demographic variables (such as gender [10]–[14], age [15], [16], weight status [17], [18] and family socio-economic status [15], [19]) were associated with physical activity in youth. From a group of 3,051 European adolescents within the age range of 12–18 years, girls were found to participate16% less in moderate and vigorous physical activity than boys [11]. Girls were also found to have a higher percentage of insufficient physical activity than boys (60% vs. 43%) in American adolescents [15]. Similarly, girls had half the amount of daily physical activity relative to boys in a sample of Taiwanese adolescents [12]. Previous studies have noted that the amount of physical activity appears to decline with age during adolescence [15], [16]. Older adolescents were reported to be more active than younger ones [10]. There is still an argument on the relationship of physical activity and age. In a comparison of overweight and obesity prevalence in school-aged children, greater participation in physical activity was associated with a lower bodyweight status in 29 out of the 34 countries investigated [20]. Other studies have found that body mass index (BMI) was positively associated with the amount of physical activity among adolescent girls [21], and that adolescents with high family socio-economic status spent more time engaging in physical activity [15], [19]. Higher education levels, both parental and maternal, are associated with higher moderate to vigorous physical activity in adolescents and higher family income was significantly positively associated with increased moderate to vigorous physical activity [15]. A previous study indicated that socio-economic status has not been found to be a determinant of activity levels in either males or females [22]. In contrast, boys with lower socio-economic status have 2–3 hours per week more moderate activities than boys with higher socio-economic status [23]. Nevertheless, gender is an important factor for physical activity whereas conflict in the continuing debate over the differences in health behaviors is due to the confounding effects of age, weight status, and socio-economic status [13], [14]. Therefore, when exploring the level of physical activity among adolescents with asthma; gender, age, weight status and socio-economic status must be taken into consideration.

More family support might produce a greater amount of physical activity in adolescents [24], [25]. Furthermore, gender differences have been found in research into family support for physical activity [26], with boys receiving significantly more family support for physical activity than girls [27]. de Moraes, Guerra, & Menezes have reported that the amount of physical activity in adolescents was lower in girls than in boys, which might be partially caused by insufficient family support for physical activity [2]. Moreover, in previous studies, family support has been regarded as an important influential factor for physical activity participation among adolescents [13], [28]. Consequently, family support should be included as a possible influential factor in the case of insufficient physical activity in adolescents with asthma.

It is important to understand the related factors (gender, age, weight status, socio-economic status and family support for physical activity) for insufficient physical activity among adolescents with asthma. Therefore, the aims of this study are to compare the level of physical activity between Taiwanese non-asthmatic adolescents versus asthmatic adolescents, as well as to investigate the influential factors associated with insufficient physical activity in Taiwan adolescents with asthma.

## Methods

### Participants

Two hundred and eighty-six adolescents with asthma and five hundred and eighty-eight non-asthmatic adolescents participated in the study. The age range was from 12 to 16 years old. After the Institute Review Board approved this study, we sent permission requests for the current study to all participants and their parents. Asthmatic children were recruited from eight junior high schools in mid-Taiwan by purposive sampling based on the following criteria: having been diagnosed with asthma by a physician at least six months prior to the study, being within the targeted age range, and not having current acute asthma exacerbations, physical limitation, or other chronic disease. Healthy children who had no asthma history, any other chronic disease, or physical limitation, and being in the same age range were randomly recruited from the same junior high schools. Parents received an Informed Consent and were informed that they and/or their children could withdraw from the study at any time. Parental permissions were signed prior to data collection.

### Demographic features

Demographic features included age, gender, height, weight, parents' occupation, parents' education, and four measures for self-perceived health status. BMI was calculated in units of kg per m^2^, and used to classify the subjects' weight status. Four groups were defined thus: underweight: <15th percentile; average weight: 15th to 85th percentile; overweight: 85th to 95th percentile; obese: >95th percentile. Cutoff points for these four groups were based on the reference data for age-and-gender-specific BMI from New Growth Charts for Taiwanese Children and Adolescents [29].

### Socio-economic status

Socio-economic status was calculated by parental occupation and education [30], and subjects were grouped into five social classes, with Social Class 1 representing the highest socio-economic status and Social Class 5 representing the lowest socio-economic status. We minimized sample size differences between cells and facilitated the comparison of the classes by grouping socio-economic status into three levels, as per the study by Oguma, et al. [31]: low (Social Classes 4 and 5), medium (Social Classes 3), and high (Social Classes 1 and 2).

### Family support

To measure family support for physical activity, we used the family part of Lee's social support for physical activity scale [32]. A higher score indicates stronger family support for physical activity. The internal reliability was 0.83, as per Cronbach's α, and test-retest reliability was 0.89 over 2 weeks (n = 48) [32]. We classified the family support distribution according to the percentile scores, classifying the subjects into two groups: low (below the 50th) and high (above the 50th).

### Physical activity amount

A Taiwanese adolescent version of the International Physical Activity Questionnaire [33] was refined from the Taiwanese version of the International Physical Activity Questionnaire [34] to measure physical activity by including five domains: activity in school, self-powered transport, household work activity, leisure time physical activity, and sedentary activity. The tool's test-retest reliability was 0.75 over 2 weeks among adolescents. Concurrent validity of the Taiwanese adolescent version of the International Physical Activity Questionnaire was 0.89 for vigorous physical activity and 0.72 for moderate physical activity, based on the 7-day physical activity recall. The correlation coefficients between RT3 measurement (which provide an estimation of energy expenditure in inactivity in adolescents) and Taiwanese adolescent version of the International Physical Activity Questionnaire was 0.55 for levels of moderate to vigorous physical activity among Taiwanese adolescents [33].

### Peak expiratory flow

The test of peak expiratory flow was measured by using a PC based flow spirometer (MIR Spirobank Spirometer Products). A trained assistant explained the purpose and technique of the test to each participant and this was followed by a demonstration of its performance. After one or two trial attempts, each participant was encouraged to make an effort and was watched to ensure that he or she maintained an airtight seal between the lips and the mouthpiece of the instrument. The best of the three consecutive measurements of peak expiratory flow values was recorded and calculated as a percentage of the predicted value based on age, height, and gender-derived norms. We classified the peak expiratory flow for severity for asthma exacerbations according to the Global Initiative for Asthma report, classifying the subjects into three groups: severe (below 60% predicted), moderate (60–80% predicted) and mild (over 80% predicted).

### Procedure

A cross-sectional study was used to investigate demographic information, weight status and social factors related to insufficient physical activity among adolescents with asthma. After being provided with a description of the procedures and purposes of the study, informed consent was obtained from all parents or guardians of the participating adolescents. All participants from eight junior high schools in central Taiwan received parental consent forms and agreed to participate in the study. We enrolled 286 adolescents with asthma and 588 non-asthmatic adolescents. Adolescents anonymously completed a questionnaire which included questions concerned with demographic information, weight status, social factors and physical activity information in school. Completed questionnaires were collected by the study assistant. The asthmatic adolescents' peak expiratory flow were obtained by the study assistant at school.

### Data analysis

SPSS for Windows (version 17.0; SPSS Inc., Chicago, IL, USA) was used to analyze the descriptive and inferential statistics. Results were presented as numbers and percentages for categorical data and were expressed as mean and standard deviation for continuous data. Chi-squared, t-test and ANOVA were performed to analyze differences in demographic data and study variables. A logistic regression model was used to determine the independent factors related to insufficient physical activity, including gender, age, weight status, socio-economic status, peak expiratory flow and family support. Statistical significance was set at α <0.05.

## Results

Table 1 displays the amount of physical activity, the family support score and the socio-demographic variables between adolescents with non-asthma and adolescents with asthma. No significant differences (p>0.05) were found for age, gender, BMI, or socio-economic status score between asthmatic and non-asthmatic adolescents. In comparison with adolescent non-asthmatics, adolescents with asthma had a higher family support score (12.2±3.9 vs. 11.0±3.9, p<0.001), greater weekly physical activity amount (671±606 vs. 507±418 min/week, p<0.001), and lower ratio of insufficient physical activity (30.8% vs. 38.6%, p = 0.02).

**Table 1:** Physical activity amount, family support score and socio-demographic variables between adolescents with non-asthma and adolescents with asthma.

Variables	Non-asthmatic adolescents (n = 588)	Asthmatic adolescents (n = 286)	
	Mean±SD	Mean±SD	p
Age (year)	13.7±0.9	13.8±1.0	.15
Body mass index(Kg/m^2^)	20.4±4.1	20.9±4.6	.12
Socio-economic status score	30.5±8.2	31.6±8.7	.07
Family support score	11.0±3.9	12.2±3.9	<.001
Physical activity amount (minutes/week)	507±418	671±606	<.001
Insufficient physical activity	227/588(38.6%)	88/286 (30.8%)	0.02
Gender(male)	346/588(59.1%)	170/286(59.4%)	.94

Insufficient physical activity, less than 300 minutes per week of moderate and vigorous physical activity for adolescents.

As shown in Table 2, there were statistically significant differences when comparing weight status, socio-economic status, family support level, and ratio of insufficient physical activity between girls with asthma and boys with asthma. The ratio of overweight and obese of boys was 25.5% higher than that of girls (p<0.001). The percentage of high socio-economic status of boys was 9.9% higher than that of girls (p = 0.02). The percentage of high family support level of boys was 21.4% higher than that of girls (p<0.001). The proportion of insufficient physical activity (less than 300 minutes per week of moderate and vigorous-intensity physical activity; MVPA300) of boys was 16.9% less than that of girls (p<0.001).

**Table 2:** Distribution of demographic data among adolescents with asthma (N = 286).

	Boys (n = 171)	Girls (n = 115)	
	n(%)	n(%)	p
Weight status			<.001
Average weight	75(45.4)	68(63.0)	
Underweight	25(15.2)	26(24.1)	
Overweight	25(14.2)	8(5.5)	
Obese	40(24.2)	5(7.4)	
Socio-economic status			.02
Low	60(37.5)	33(33.0)	
Medium	73(45.6)	60(60.0)	
High	27(16.9)	7(7.0)	
Family support level			<.001
Low	69(40.4)	71(61.7)	
High	102(59.6)	44(38.2)	
PEF(% predicted)			.07
>_ = _80	135(81.8)	97(89.8)	
60–80	30(18.2)	11(10.2)	
Insufficient physical activity			.002
Yes	41(24.0)	47(40.9)	
No	130(76.0)	68(59.1)	

Weight status: underweight, BMI <15th percentile; average weight, BMI 15th to 85th percentile; overweight, BMI 85th to 95th percentile; and obese, BMI >95th percentile.

Socio-economic status: high, Social Classes 1 and 2 of Hollingshead's Socio-economic status; medium, Social Classes 3 of Hollingshead's Socio-economic status; low, Social Classes 4 and 5 of Hollingshead's Socio-economic status.

Family support level: low, below the 50th of the percentile scores; high, above the 50th of the percentile scores.

PEF, Peak expiratory flow.

Insufficient physical activity, less than 300 minutes per week of moderate and vigorous physical activity.

Table 3 compares the amount of physical activity and insufficient physical activity. Girls had a higher ratio of physical inactivity (less than 300 minutes per week of moderate and vigorous-intensity physical activity; 40.9% vs. 24%) but less moderate and vigorous physical activity than boys (484±438 vs. 797±668 minutes/week). Adolescents with low family support showed a higher ratio of physical inactivity (less than 300 minutes per week of moderate and vigorous-intensity physical activity; 45% vs. 17.1%) and less moderate and vigorous physical activity than those with high family support (504±514 vs. 831±646 minutes/week). Weight status presented a borderline relationship with the ratio of insufficient physical activity (p = 0.05) and no significant difference with moderate and vigorous physical activity.

**Table 3:** Comparison of the amounts of physical activity and insufficient physical activity (n = 286).

	Amount of moderate and vigorous physical activity			*p*	Number of Insufficient physical activity	*p*
	(minutes/week)	Median	25^th^–75^th^ percentile		(n/total n of subgroup)	
Gender				<0.001		.002
Boys	797±668	580	300-1175		41/171	
Girls	484±438	370	185–645		47/115	
Weight status				.44		.05
Average weight	728±543	435	210–820		52/143	
Underweight	646±653	577	332–1044		9/51	
Overweight	837±692	540	260–997		11/33	
Obese	673±488	615	300–1307		10/45	
Socio-economic status				.55		.12
Low	776±572	645	337–984		31/93	
Medium	714±610	480	265–1030		38/133	
High	636±625	427	224–902		5/34	
Family support				<0.001		<0.001
High	831±646	617	340–1164		25/146	
Low	504±514	324	140–654		63/140	
PEF(% predicted)				.32		.34
> = 80	655±653	435	220–925		74/232	
60–80	762±619	570	231–1337		10/41	

Weight status: underweight, BMI <15th percentile; average weight, BMI 15th to 85th percentile; overweight, BMI 85th to 95th percentile; and obese, BMI >95th percentile.

Socio-economic status: high, Social Classes 1 and 2 of Hollingshead's Socio-economic status; medium, Social Classes 3 of Hollingshead's Socio-economic status; low, Social Classes 4 and 5 of Hollingshead's Socio-economic status.

Family support level: low, below the 50th of the percentile scores; high, above the 50th of the percentile scores.

PEF, Peak expiratory flow; insufficient physical activity, less than 300 minutes per week of moderate and vigorous physical activity.

Using unadjusted and adjusted logistic regression model analyses, the variables related to insufficient physical activity among adolescents with asthma are presented in Table 4. In the unadjusted analysis model, boys were less likely to be insufficiently active (95% CI = .27–.76), and obesity was related to insufficient physical activity (95% CI = 1.08–6.67). Adolescents with high family support were less insufficiently active than those with low family support (95% CI = .15–.43). The other variables were not significantly related to insufficient physical activity in the unadjusted analysis model. After adjustment, girls, obesity and low family support were related to insufficient physical activity.

**Table 4:** Insufficient physical activity among adolescents with asthma (n = 286).

Variables	COR(95% CI)	*p*	AOR(95% CI)	*p*
Gender		.003		.01
Girls (ref)	1		1	
Boys	.46(.27–.76)		.41(.21–.81)	
Weight status		.11		.13
Average weight (ref)	1		1	
Underweight	2.22(.69–7.14)	.18	2.73(.77–9.70)	.12
Overweight	1.30(.42–4.00)	.65	1.79(.53–6.13)	.35
Obese	2.63(1.08–6.67)	.03	2.99(1.15–7.75)	.02
Socio-economic status		.25		
Low (ref)	1			
Medium	2.17(.68–6.67)	.19		
High	2.70(.83–8.33)	.10		
PEF(% predicted)		.50		
<80 (ref)	1			
> = 80	.74(.30–1.79)			
Family support level		<0.001		0.004
Low (ref)	1		1	
High	.25(.15–.43)		.39(.20–.74)	
Age		.33		
12–13 (ref)	1			
14–16	1.30(.76–2.22)			

Weight status: underweight, BMI <15th percentile; average weight, BMI 15th to 85th percentile; overweight, BMI 85th to 95th percentile; and obese, BMI >95th percentile.

Socio-economic status: high, Social Classes 1 and 2 of Hollingshead's; medium, Social Classes 3 of Hollingshead's Socio-economic status; low, Social Classes 4 and 5 of Hollingshead's Socio-economic status.

Family support level: low, below the 50th of the percentile scores; high, above the 50th of the percentile scores.

PEF, Peak expiratory flow.

Insufficient physical activity, less than 300 minutes per week of moderate and vigorous physical activity.

COR, crude odds ratio.

AOR, adjusted odds ratio.

## Discussion

The main findings in the present study are (1) adolescents with asthma in Taiwan had a lower ratio of insufficient physical activity, and a greater amount of weekly physical activity than non-asthmatic adolescents, (2) The significant associated factors of insufficient physical activity among asthmatic adolescents in Taiwan are; girls, obesity and low family support.

Asthmatic adolescents are usually less active than their non-asthmatic peers with suffering from asthma being given as the reason for lower physical activity, in an asthmatic physical activity review [35]. Similar to our findings, Weston et al. found asthmatic adolescents were significantly more active than non-asthmatic adolescents [36]. Another systematic review has reported that, in many studies, there are no significant differences in physical activity between asthmatic children and non-asthmatic children, and suggested that high physical activity levels may protect against the development of asthma [37]. Chiang's study pointed out that asthma intervenes in children's capability to participate in vigorous physical activity but not in moderate to vigorous physical activity [7]. In this Taiwan-based study we found that adolescents with asthma had a lower ratio of insufficient physical activity, a higher level of physical activity, and higher family support than non-asthmatic adolescents. Since parental concerns and support could be the reason for improving physical activity [26], our study suggests that a higher level of family support might be an influential factor in causing a lower ratio of insufficient physical activity in asthmatic adolescents than non-asthmatic adolescents.

Our study found a gender difference among adolescents with asthma, namely that boys were more likely to engage in physical activity. Indeed, our study found that 40.9% of the adolescent girls cannot achieve the global requirements for physical activity, whereas only 24% of the adolescent boys cannot achieve the requirements. Gender differences in the physical activity of adolescents remain the most studied of the demographic variables [13], [14]. The determinants of adolescents' physical activity behaviors seem to differ significantly by gender. Two possible reasons why there might be differences in physical activity by gender could be; maturity variation and different societal roles. Adolescent girls are fatter in body composition, broader in hip proportions, and less proficient in motor skills as well as periodically suffering from menstrual discomfort and lower blood hemoglobin levels [38]. In societal roles, Taiwanese adolescents often believe that boys should be brave and strong and should participate in sports activities, whereas girls should be tender and friendly [39]. Therefore, different socialization patterns for boys and girls may restrict the opportunities and the accessibility of certain types of physical activity.

Family support is an important factor in the social environment which influences physical activity behaviors [13], [28]. Physical activity of adolescents is associated with the parental exercise habit, family support for physical activity, and parent-child relationship [40]. In this study, asthmatic adolescents with higher family support appeared to be more physically active than those with lower family support. Teenagers' parents play important roles in promoting their physical activities, so we rationally expected that family support would encourage a healthier approach to physical activity among adolescents with asthma. The findings of this study further support adolescents' physical activities having a positive association with family support [25]. The results show that boys had higher levels of parental support for physical activity than girls [24]. The girls, on the other hand, had more barriers to physical activity and disliked being active, which might follow from gender stereotypes in Taiwan. Parental support for physical activity (such as participating in physical activity and transporting them to activities) is also highly associated with adolescent participation in physical activity [41]. In other words, parental support for physical activity comes in many forms, such as parental verbal encouragement, parent transportation, parent payment of fees for physical activity, etc. Therefore, physical activity promotion interventions could target the needed types of family support for adolescents.

Adolescents with asthma were more likely to be obese and were significantly less active than non-asthmatic adolescents in the United Kingdom [42]. The symptoms; shortness of breath, coughing, chest tightness, and wheezing might present in the course of exercise challenge and thus adolescent asthmatics may avoid physical activities for fear of exacerbating their asthma [43]. Therefore, some adolescents with asthma and their parents recognized asthma as a medical excuse for physical inactivity [42]. _ENREF_47However, Eijkmanset al. did not support that wheezing children or children with asthmatic symptoms had a lower level of physical activity or were more overweight [44]. Overweight children and adolescents reported more fractures and musculoskeletal discomfort (21.4% overweight vs. 16.7% non-overweight), which suggests that orthopedic difficulties may partly give rise to less physical activity and an accumulation of excess weight in children [45]._ENREF_49 Our present study found that obese adolescents with asthma were significantly less active than normal weight children with asthma in Taiwan, but not in overweight adolescents. _ENREF_49_ENREF_48The possible reasons maybe greater parental support for physical activity in overweight adolescents with asthma, and being overweight might not be a limiting factor for physical activity.

There are several limitations to this study. First, there is the limitation that this study is used a cross-sectional design and therefore it is difficult to establish a causal relationship between family support and physical activity. Secondly, self-reported information in the present study could be less reliable than standardized devices in detecting physical activities. Third, though our study focused on adolescents with asthma, their peak expiratory flow was above the 60% predicted in our study. Therefore, the results of this study are only applicable to above 60% predicted peak expiratory flow adolescents. This also precludes the ability to make new conclusions as to how the health status of asthma sufferers affects their physical inactivity. Consequently, objective indicators and educational intervention can be applied in future studies.

In summary, adolescents with asthma had 30.8% insufficient physical activity. Furthermore, the predictors of insufficient physical activity are gender, obesity and family support among adolescents with asthma in Taiwan. Programs to remedy insufficient physical activity should facilitate family support and give special attention to girls, and the obese among adolescents with asthma.
